# Substance of a Letter to the Commissioners for (Transports, and) Sick and Wounded Seamen, upon the Utility of Stimulants, and the Impropriety of Warm Cataplasms as General Applications in the Treatment of Ulcers

**Published:** 1810-05

**Authors:** D. I. H. Dickson

**Affiliations:** Physician, and Inspector of his Majesty's Fleet, at the Leeward Islands


### *Substance of a Letter to the Commissioners for (Transports, and) Sick and Wounded Seamen, upon the Utility of Stimulants, and the Impropriety of warm Cataplasms as general Applications in the Treatment of Ulcers.* By D. I. H. Dickson, M. D. Physician, and Inspector of his Majesty's Fleet, at the Leeward Islands.

THE consideration of Ulcers, which in my letter to you of the 1st of August, I expressed my intention of resuming, is of much greater importance than probably may be supposed by those who have not had extensive opportunities of witnessing opposite modes of treatment, and thereby of judging of the accumulated sufferings of the loss of limbs, and even of lives, that may accrue from improper treatment. A diffuse view of a subject upon which so much has been written, would be altogether superfluous; and without meaning to discuss the nature and varieties of ulcers as they have occurred in different climates, in unhealthy healthy situations, from unwholesome diet, or as connected with constitutional disease; my object is merely to recommend, generally, the use of stimulants for their cure, and to point out the pernicious effects of warm cataplasms and emollient greasy applications. Besides many others who have noticed the bad effects of such remedies, Dr. Trotter, in treating of the malignant Channel ulcer, remarks, that cold applications in the inflammatory stage, seemed to answer better than fomentations. These ulcers, he observes, extended rapidly over the limb, with a profuse fœtid discharge, and left the bone almost bare its whole length. From whatever causes this might arise in the Channel, effects very similar are apt to be produced by warm poultices in the West Indies. Their application is succeeded (in proportion to the tendency of the ulcer to malignancy) by a profuse, vitiated discharge, with a loss of substance so rapid, that one might almost imagine the fleshy parts are seen melting away, or disappearing before the eyes; and thus a small sore, in a few days, becomes a painful, extensive, foul, sloughing ulcer. That this has lately been strikingly illustrated, will appear by the following extract of a report made to me by Mr. Moore, late surgeon of the Marine garrison at Mariegalante, dated July 3, 1808. After stating that there were sixty-five cases of ulcer then in the sick report, he observes:

"Prior to my illness we had scarcely a case shewing a tendency to sloughing; but during the nine days that Mr. —— had charge of my sick, spirits and other stimulants (precisely after the manner you recommended to Mr. Nesbitt, and in which practice I had with success treated my ulcers) were thrown aside, and poultices substituted in their place, not only in the hospitals, but in the trifling sores of such as were not in the sick list, (always numerous). The consequence was, as you cautioned Mr. Nesbitt; sores, before inconsiderable, became sloughing ulcers. These cases crowded in day after day, and from not having an hospital sufficiently airy, and large, &c. it is no wonder that they soon became the completely contagious, sloughing ulcer. Lately, better accommodations have been procured as to air, &c. and with evident good effects. Of the cases now reported, twenty are in a sloughing state, forty-five granulating, and some nearly healed."

Previously to the receipt of this Report, I had learnt the great increase of ulcers in that garrison; and suspecting some impropriety of treatment, I had written to Mr. Nesbitt, the successor of Mr. Moore, recommending stimulants, the the total omission of cataplasms, attention in dressing, and the separation of the bad cases from the benign. On my arrival soon after at Mariegalante, at the end of July, tho' I found the general sickness much increased, and 75 ulcers on the list, yet many of them had much improved by the means above recommended. "Some were still extremely malignant; but the greater part were well conditioned and healing." Mr. Nesbitt was taken ill, and the ulcers were degenerating into highly malignant sphacelating stages, when he was relieved for a while by Mr. Mortimer, surgeon of the flag ship, by whose attention and assiduity they were brought into a state of immediate and rapidly progressive improvement. He informed me, the only means he employed were great attention in cleaning the sores, the application of rum, and compression. Mr. Mortimer was also taken ill, and the ulcers, under a French practice of unguents, &c. were again getting rapidly worse, when he was relieved by Mr. Waller, under whose treatment, consisting entirely of stimulants, they were again improving by the last accounts which I have received. Under all these disadvantages, and amidst great sickness and mortality, only two deaths can be traced to this source, and there are two or three cases of caries which may require amputation; but if warm poultices, and emollient ointments had been persisted in throughout, what would have been the scene of misery; what would have been the loss of limbs and of lives from this cause alone, it is painful to conjecture.

The Board will recollect that a similar instance occurred on board of his Majesty's ship the Northumberland, off Ferrol, in 1804. That ship had been almost over-run with ulcers, when the late Dr. Stephen joined. He "found forty-three ulcers on the medical list, most of which appeared to be either in a state of high irritation and inflammation, or inactive, and torpid; with marks of gangrene and sphacelus." By the entire prohibition of cataplasms, and by the use of stimulants, they were reduced to six in a short time, and these were in a fair way of recovery, as stated by him to the Board, in a letter dated December 19, 1804. These evidences bear so decisively on the point in question, that I believe it unnecessary to bring forward any of the reports that have been made to me on ulcers on this station, or to detail the success of the stimulating plan in the hospitals here, with which you must be well acquainted. But exceptions must ever qualify general approbation; probation; it is not pretended that spirit or other stimulants will arrest the progress of sphacelus in ulcers, but they will assist the extrusion, and greatly lessen the fœtor of the diseased parts; while in the simple ulcer, they will hasten cicatrization; and in the vitiated, they will tend to alter or destroy the morbid action going on.

Referring to my own experience, I have not found much difficulty in curing the general run of ulcers, or, at least in preventing their gaining any ascendancy; when I have failed in these objects, they have been of long standing, and had not been properly attended to in their early stages. I have, indeed, seen shocking cases of ulcers, but I am convinced that these had either been greatly neglected at first, or had been improperly poulticed, or had been plastered with ointments, or pasted up with various powders, until the confined and vitiated discharge became more and more morbid, and specifically malignant. This must be more especially the case in warm climates: hence the importance of dressing sores at least twice a day in such situations, of cleaning them well, and of changing the action of the vessels that secrete the morbid matter by stimulants. The remedies of this description, from which I have seen the most benefit, are diluted alcohol, or common proof spirit, as rum, brandy, &c. Solutions of the nitrate of silver (of various degrees of strength, but on an average, 12 grains to an ounce of water), of the sulphate of copper, red oxide of mercury, lime or lemon juice, &c. The latter, which has been strongly recommended by Drs. Blane and Gillespie, is very generally used, and with excellent corrective effects in foul, dark coloured, fœtid, or scorbutic ulcers. But as applications of general utility and efficacy, I prefer lint dipped in proof spirit, or the solution of the nitrate of silver, to the others. This is, however, of little consequence; as the certainty and quickness with which the ulcer may be cured, depend much upon changing the stimulus. When one remedy of this class has been long persisted in, it loses its effect, and the ulcer ceases to improve; in this case it should be immediately susperseded by another, and that again by a third, and so on, recurring afterwards to the first, when it will be found to have regained its original influence. The benefit of this plan has been so generally noticed of late, that I trust it is superfluous to insist upon it farther.

A tight, well applied bandage of calico, will also generally much contribute to the cure; and where there is surrounding, slow, deep-coloured inflammation, it should be be frequently wetted with spirit, lemon juice, saturnine solution, or water. It is often remarkable, how speedily the redness, tumour, and pain are reduced by these remedies, which act by increasing and supporting the energy and contractile power of the vessels, and thus assisting them to transmit the accumulated blood, by which, in this inactive kind of inflammation, they were distended and oppressed; while, on the other hand, warm applications will farther solicit determination, and increase the atony and relaxation of the diseased parts. Finally, then, my opinion of warm poultices is explicitly this: there are, occasionally, states of ulcer where gentle warmth and moisture, through the medium of a few cataplasms, will produce a soothing and cleansing effect; and there are others, where the healthy progress is so strong as not to be easily perverted by any application, however untoward; but with these exceptions, I infer that their temporary use is generally inexpedient, in warm climates at least; and that their continuance is always highly hurtful. When an ulcer has required a change of appearance, they have been too often resorted to without sufficient attention or discrimination. Every practitioner has recurred to them, and has been obliged to leave them off repeatedly; while many, if they have employed stimulants at all, and the ulcer did not instantly improve, or particularly, if it seemed to degenerate, have thrown them aside, condemned after a few inadequate trials. Should the employment of stimulants so very generally, if not exclusively, appear too indiscriminate, I have only to remark, that I have not found this to be the case, while I have seen much of the bad effects of the practice, which I here conceive it my duty to reprobate.

Of the difficulty of curing ulcers, in all ages, in certain situations and habits, or particularly on the lower extremities, I am perfectly aware; without going farther back, Baglivi states that ulcers on the legs were almost incurable at Rome, and wounds difficult to heal; while the like on the head were easily cured. Drs. Cleghorn and Hunter observed the same thing at Minorca and Jamaica; and very many authorities testify the great obstinacy of ulcerated legs, particularly in warm climates, and often the impossibility of curing them at all; yet, that they would frequently heal on the passage homeward. But as this difficulty, as well as the tendency to recur, will greatly depend on the length of standing of ulcers, which will again depend depend on the manner they are treated at first, I must still express my opinion, that from the improved victualling of the navy, and from the improved method of treatment, most ulcers can now be certainly cured, the generality easily; or at any rate prevented from becoming malignant, in the ordinary course of service at least. The cause of the frequent return of ulcers has been too little attended to, but is of such importance, that I trust to be excused repeating how much it is influenced by the plan of cure. When this has been weak, inactive, or improper, a cure, if at all obtained, is so long in taking place, that a habit is acquired, facilitating the recurrence of this evil from the most trivial causes: hence I have observed, that the same men have been put into the medical list, again and again, in the course of the year; and a great majority of ulcerated patients are ever found on inquiry to have had ul cer before. In this respect, ulcer is similar to dysentery; where these diseases have been of long standing, or have recurred two or three times in this country, they should be sent home. Yet these cases often seem trifling, and not unfrequently get well, or so far improve during the passage, as on their arrival to appear to have been invalided without sufficient reasons.

The state of the body has been too little attended to during the cure of ulcers. The digestive organs, the regularity of the bowels, and general habit, should be particularly enquired into, and any aberations corrected by aperients, tonics, stimulants, and alteratives. The importance of this, and the reciprocal influence of diseased actions are so fully illustrated in Mr. Abernethy's Treatise on "The Disorders of Health," as to preclude any farther observations here. Without taking upon me to decide upon the points of identity or diversity, in specific or particularly malignant, sphacelating, or obstinate ulcers, such as the phagedæna, and hospital sore, of Drs. Adams and Rollo; and those described by Drs. Trotter, Harness, Gillespie, &c. in the Channel, Mediterranean, East and West Indies; and without citing the experiments that have been made on healthy pus by Messrs. Hunter, Home, and many others, as contrasted with the specific, morbid discharges, examined by Drs. Crawford, Mitchell, Mr. Cruikshank, &c. I here merely beg leave to refer to those celebrated authorities, because from such a review I apprehend much may be found, or inferred, in favour of stimulating or active remedies.

The

The fœtor of the hospital sore, Mr. Cruikshank found was most effectually destroyed by the nitrate and muriate of mercury, by the nitrous acid, and especially by the oxygenated muriatic acid, in the form of liquid, or gas; and Dr. Rollo was most successful in curing the same ulcer by these remedies, and the nitrate of silver. Dr. Mitchell treated foul, ill-conditioned ulcers with alkalies, with great success; and his theory of acidity, which led to their employment, whether admitted or not, does not affect their powerfully stimulating property. Dr. Underwood, I believe, recommends stimulants generally, for the cure of ulcers. The good effects of such applications in extremely painful and irritable venereal sores, might also be adduced in favour of this practice. The application of nitre to ulcers, highly extolled by the late Dr. Cuming, I find was used in the naval hospital at Halifax, many years ago; and I was there informed, it was first tried by Dr. Paterson at the Cape of Good Hope. As it gives intense pain, and is mentioned without praise, I think it may well give way to other powerful and less distressing remedies. I have no experience of its effects in gangrene. Some writers, and particularly Mr. Whately and Dr. Underwood, have strongly recommended exercise during the cure of ulcers, while rest and a horizontal position of the limb, have been inculcated by the majority of practitioners, and especially by my much lamented friend, the late Mr. Benjamin Bell. The advantages of each of those modes in different states of ulceration, cannot, I think, be questioned; but generally speaking, I must give a decided preference to rest, because I have so very often found the cure greatly retarded, or altogether prevented, while men were permitted to walk about. In the active, inflammatory, or progressive stages, it is indispensable. By an horizontal position, and a cooling regimen, ulcers tending to malignancy may often be subdued in the beginning; while, on the other hand, in the chronic, torpid stage of ulceration, exercise may not only be innocuous, but in many cases highly beneficial, by improving the local and general health, and vigour of circulation. Neither the theory of a dependant posture, nor of a defect of vital energy, can therefore affect the propriety of exercise, or rest, in opposite states of ulcers.

I am happy to find my ideas on the treatment of ulcers in coincidence with those of a very able practitioner, Mr. M'Arthur, of the naval hospital at Barbadoes, who has transmitted to you a most valuable paper on this subject, the result of much practical observation, and to whose recommendation, and diffusion of the good effects of spirituous and other stimulating applications, the cure of ulcers in the West Indies is much indebted.

*Sept.* 1, 1808.

